# Predictors of poor medication adherence in patients undergoing peritoneal dialysis: a LASSO-based risk model from a cross-sectional study in Xinjiang, China

**DOI:** 10.1080/0886022X.2026.2687196

**Published:** 2026-06-24

**Authors:** Hui Liu, Hong Tan, Li Li

**Affiliations:** ^a^ Department of Urology, The First Affiliated Hospital of Xinjiang Medical University, Urumqi, China; ^b^ School of Nursing, Xinjiang Medical University, Urumqi, China; ^c^ Health Care Research Center for Xinjiang Regional population, Urumqi, China

**Keywords:** China, maintenance peritoneal dialysis, medication adherence, self-management, helplessness, LASSO logistic regression model

## Abstract

Medication nonadherence is a major challenge in peritoneal dialysis, yet predictive tools are available. This study aimed to identify key determinants of poor medication adherence and develop a clinically applicable prediction model. A cross-sectional survey was conducted among 401 patients undergoing maintenance peritoneal dialysis in Xinjiang, China. Data included sociodemographic characteristics, medication and medical history, and validated scales for self-management, self-efficacy, and helplessness. Least absolute shrinkage and selection operator (LASSO) logistic regression with 10‑fold cross-validation was used to select predictors and construct the model; model performance was assessed using the area under the curve (AUC), Brier score, and calibration curve. Overall, 286 participants (71.3%) had poor medication adherence. The LASSO model (λ = 0.054) retained three predictors: medication side effects, self-management level, and helplessness. Compared with the full‑variable model, the LASSO model achieved lower AIC (370.19 vs. 371.95) and BIC (386.17 vs. 399.91), indicating greater parsimony. The stepwise model had a slightly lower AIC, but its BIC was higher. Discrimination and calibration were comparable across models. Poor adherence is highly prevalent and primarily associated with medication side effects, inadequate self-management, and helplessness. The LASSO‑based model provides a concise, interpretable tool for identifying high‑risk patients to support targeted interventions.

## Background

Chronic kidney disease that progresses to the end stage results in irreversible damage to kidney function and structure, at which point patients can only rely on renal replacement therapy (including kidney transplantation, hemodialysis [HD], and peritoneal dialysis [PD]) to sustain their lives [1]. PD has gradually become the preferred treatment for patients with end-stage renal disease due to its safety and effectiveness, its home‑based nature, and its ability to preserve residual kidney function [2]. According to statistics, PD accounts for 11% of all dialysis cases worldwide and 9% of all renal replacement therapies [3]. In 2018, China’s national blood purification case information registration system indicated that over 90,000 individuals chose PD, accounting for approximately 16.4% of all dialysis patients [2]. However, PD patients often face complications, such as malnutrition, electrolyte metabolism disorders, hyperlipidemia, and hyperuricemia [4,5], which require long-term or even lifelong treatment with multiple medications. Medication adherence directly affects patient prognosis and disease progression [6]. Nevertheless, the factors influencing medication adherence are numerous and complex. Accurately identifying these factors and implementing timely, effective interventions to reduce the risk of disease progression are of great clinical importance. At present, research on medication adherence among PD patients remains limited both in China and internationally. Although existing studies have reported on this topic, their sample sizes are small, typically ranging from 82 to 235 cases [7–9]. Therefore, this study aims to explore the key factors of poor medication adherence in a large sample of PD patients and to establish a predictive model. The findings will provide a theoretical basis for personalized medication management in patients undergoing maintenance PD.

## Methods

This study was reported in accordance with the Strengthening the Reporting of Observational Studies in Epidemiology (STROBE) checklist for cross-sectional studies [10] (Appendix 1).

### Design

This study was a cross-sectional survey.

### Setting and sample

A total of 401 patients who underwent regular dialysis at the maintenance peritoneal dialysis centers of the First Affiliated Hospital of Xinjiang Medical University and the People’s Hospital of Xinjiang Uygur Autonomous Region between October 2022 and May 2023 were selected as the study subjects.

### Inclusion criteria

(1) Age > 18 years; (2) dialysis duration of no less than 3 months; and (3) voluntary participation in this study and signed informed consent.

### Exclusion criteria

(1) Communication barriers; (2) concurrent tumors or critical illness.

### Sample size

According to the standard sample size calculation for cross-sectional surveys [11], the sample size should be 10–20 times the number of study variables. In this study, the variables comprised 16 items from the general information questionnaire, one each from the Morisky Medication Adherence Scale and the General Self‑Efficacy Scale, six from the self‑management scale for patients on continuous ambulatory PD, and two from the Chinese version of the Learned Helplessness Scale for Maintenance Hemodialysis (MHD) Patients, giving a total of 26 variables. Allowing for a 20% sample attrition, the final estimated sample size ranged from 325 to 650.

### Recruitment

The electronic questionnaire was created using Wenjuanxing (https://www.wjx.cn/), a professional online survey platform in China [12]. The survey was distributed *via* WeChat, a widely used social media application in China. All questionnaire items were uploaded to Wenjuanxing, which then generated a website link and a QR code for easy access to the survey. The research team obtained approval for this study from the directors of the clinical departments and the hospital administration. Trained investigators informed potential participants about the purpose, content, and requirements of the study and distributed the website link and QR code of the online questionnaire to them through WeChat. The participants could access the questionnaire by clicking the link or scanning the QR code with their mobile phones. The anonymity of the participants’ responses and the confidentiality of their personal information were ensured, and participation in the study was entirely voluntary. Questionnaires completed in less than 5 min or more than 60 min were considered invalid.

### Variables and measurements

The online questionnaire included a general information form, the Chinese version of the Morisky Medication Adherence Scale (C-MMAS-8), the Self-Management Ability Scale (SMAS), the General Self-Efficacy Scale (GSES), and the Scale of Learned Helplessness in MHD Patients (Chinese Version) (LHS-MHD-C). The questionnaire used in this study was specifically designed for this research and has not been previously published. An English-language version of the questionnaire is provided as a supplementary file (Supplementary Material 1).

### General information survey form

The researchers independently developed the criteria, which included biologic sex, age, ethnicity, marital status, educational level, occupational status, type of medical insurance, duration of dialysis, dialysis operator, dialysis treatment situation, average number of hospitalizations per year, number of medications taken, and number of medication side effects.

### C-MMAS-8

The MMAS was initially developed by Morisky [13] and has been translated into many languages by scholars, including Chinese [14] (©MMAS 2006 used with permission www.adherence.cc). The C‑MMAS‑8 consists of eight items, of which questions 1 ∼ 7 are scored as 1 point for ‘no’ and 0 points for ‘yes’. Question 8 is scored as 0, 0.25, 0.5, 0.75, and 1 point for responses of ‘very difficult’, ‘difficult’, ‘average’, ‘easy’, and ‘very easy’, respectively. The total score ranges from 0 to 8 points. Patients were divided into two categories according to their score: poor adherence (score <6 points) and good adherence (score ≥ 6 points).

### SMAS

This scale was developed by Pang Jianhong [15] and includes six dimensions: fluid exchange technology operations (7 items), handling of abnormal situations (4 items), dietary management (5 items), monitoring of complications (8 items), emotional management, and social reintegration (4 items), for a total of 28 items. It uses a 4‑point Likert scale (0–3 points), and the total score ranges from 0 to 84 points. A high score indicates strong self‑management ability.

### GSES

This scale was developed by Scbwanter et al. and was translated into Chinese by Wang Caikang [16] in 2000. It has been used in various fields by the academic community to assess individuals’ self-efficacy. This scale is unidimensional, consisting of 10 items, each rated from 1 to 4 points based on the responses ‘completely incorrect’, ‘somewhat correct’, ‘mostly correct’, or ‘completely correct’. A high score indicates high self‑efficacy.

### Lhs-mhd-c

Chinese scholars Xie Chunyan et al. [17] obtained this scale after completing the cross-cultural adaptation of the Arthritis Helplessness Index. The scale includes two dimensions and 11 items and uses a 5-point Likert scoring method (1–5), with a total score ranging from 11 to 55. A high score indicates a high level of learned helplessness.

### Data collection

The questionnaire survey was administered by trained, responsible nurses. Before the survey began, the nurses provided patients with a detailed face‑to‑face introduction to the questionnaire completion guidelines and obtained their informed consent. The nurses then sent the patients a link to the electronic questionnaire, encouraging them to complete it independently to ensure data authenticity and reliability. For patients who had difficulty completing the questionnaire independently due to comprehension issues or other reasons, the responsible nurses provided necessary explanations and assistance. After the electronic questionnaire was completed, the researchers promptly exported the data and conducted a rigorous quality check to ensure the accuracy and completeness of the responses.

### Data analysis

The traditional regression model uses the least squares method for unbiased estimation of the dataset [18]. However, when analyzing the many factors contributing to poor medication adherence among PD patients, the traditional regression model is prone to overfitting due to model complexity. Multicollinearity among the factors can also affect the stability of the model. LASSO regression, which is also based on the least squares method, adds the sum of the absolute values of the regression coefficients as a penalty term, shrinking the coefficients of independent variables that have a minimal relationship with the dependent variable to zero, thereby achieving model sparsity. Although it introduces some estimation bias, it effectively selects variables [19]. Furthermore, LASSO regression addresses overfitting and multicollinearity in traditional regression models, simplifies the model structure, and enhances predictive accuracy and generalizability, making the model easy to apply [20].

Data analysis was conducted using SPSS version 22.0 and R version 4.4.2 software. Normally distributed quantitative data were presented as mean ± standard deviation, and between‑group comparisons were performed using the independent‑samples t-test. Non‑normally distributed quantitative data were presented as median with interquartile range [M (IQR)], with between‑group comparisons conducted using non‑parametric tests. Categorical data were presented as counts (%), and between‑group comparisons were performed using the chi‑square test. The LASSO logistic regression model was constructed using the ‘glmnet’ package in R software, and the optimal tuning parameter λ was selected through cross-validation. This model was compared with the full-variable logistic regression and stepwise logistic regression (α in = 0.05, α out = 0.10) in terms of AIC and BIC to assess model fit. The AUC, Brier score, and calibration curves were used to evaluate the predictive performance of the models. The ‘rms’ package in R software was employed to present the model nomogram and calibration curves for the models.

### Ethical considerations

This study was approved by the hospital ethics committee (K201908‑06). All procedures were conducted in accordance with the principles outlined in the Declaration of Helsinki. Prior to the study, the research team obtained permission from hospital management and the human resources department. Written informed consent was obtained from all participants. An electronic informed consent form was displayed on the cover page of the online questionnaire, and participants were required to click ‘I agree to participate’ before proceeding to the questionnaire. Participation was entirely voluntary, and all participants were informed of their right to withdraw from the study at any time without consequences. All data collected from the participants were kept confidential and anonymized to ensure privacy protection.

## Results

### Demographic characteristics of participants

This study enrolled 401 PD patients, including 231 males (57.6%) and 170 females (42.4%), with a mean age of 45.72 ± 13.33 years. According to the medication adherence assessment, 286 participants (71.3%) were classified as having poor medication adherence.

### Comparison of medication adherence among PD patients with different characteristics

Univariate analysis showed a statistical association (*p* < 0.10) between medication adherence and the following variables: marital status, PD status, PD operator, medication side effects, self-management, and helplessness, as shown in Table 1.

**Table 1.** Univariate analysis of medication adherence among PD patients.

Characteristics	Low adherence (*n* = 286)	Moderate/high adherence (*n* = 115)	*X*^2^/*t*/*Z*	*p*
Sex			0.526	0.503
Male	168 (58.7%)	63 (54.8%)		
Female	118 (41.3%)	52 (45.2%)		
Age			2.306	0.316
<45 years	131 (45.80%)	48 (41.74%)		
45–64 years	131 (45.80%)	61 (53.04%)		
≥65 years	24 (8.4%)	6 (5.2%)		
Education level			1.025	0.611
Primary school and below	45 (15.7%)	16 (13.9%)		
Middle school	137 (47.9%)	51 (44.3%)		
College and above	104 (36.4%)	48 (41.8%)		
Marital status			8.677	0.013**
Unmarried	53 (18.5%)	15 (13.0%)		
Married	205 (71.7%)	97 (84.4%)		
Divorce	28 (9.8%)	3 (2.6%)		
Ethnicity			0.029	0.906
Han	194 (67.8%)	77 (66.9%)		
Other	92 (32.2%)	38 (33.1%)		
Employment status			1.450	0.254
Employment	101 (35.3%)	48 (41.7%)		
Other	185 (64.7%)	67 (58.3%)		
Type of medical insurance			0.244	0.915
Self-funded	7 (2.5%)	3 (2.6%)		
Urban Residents Medical Insurance	218 (76.2%)	90 (78.3%)		
New Rural Cooperative Medical System	61 (21.3%)	22 (19.1%)		
Household registration nature			1.110	0.304
Rural area	108 (37.8%)	37 (32.2%)		
Town	178 (62.2%)	78 (67.8%)		
Peritoneal infection			0.953	0.379
Yes	71 (24.8%)	34 (29.6%)		
No	215 (75.2%)	81 (70.4%)		
PD status			14.009	0.001***
Continue PD	234 (81.8%)	92 (80.0%)		
Do not continue PD	17 (5.9%)	18 (15.7%)		
Alternating between PD and HD	35 (12.3%)	5 (4.3%)		
Dialysis age			1.088	0.581
<12 months	79 (27.62%)	36 (31.30%)		
12–36 months	86 (30.07%)	29 (25.22%)		
≥36 months	121 (42.31%)	50 (43.48%)		
PD operator			3.546	0.074*
Self	257 (89.9%)	110 (95.7%)		
Family member	29 (10.1%)	5 (4.3%)		
Types of medication			1.719	0.430
≤2 medications	26 (9.1%)	12 (10.4%)		
3–4 medications	135 (47.2%)	46 (40.0%)		
≥5 medications	125 (43.7%)	57 (49.6%)		
Side effects of medication			23.214	<0.001***
0–1	133 (46.5%)	83 (72.2%)		
2–3	116 (40.6%)	28 (24.3%)		
>4	37 (12.9%)	4 (3.5%)		
Number of hospitalizations per year			1.151	0.562
1–2	189 (66.1%)	76 (66.1%)		
3–5	80 (28.0%)	29 (25.2%)		
>6	17 (5.9%)	10 (8.7%)		
Medication Guidance			1.131	0.298
Need	183 (64.0%)	80 (69.6%)		
No need	103 (36.0%)	35 (30.4%)		
Self-management	61 (49.75, 72.25)	71 (58, 78)	−4.798	<0.001***
Self-efficacy	24 (20, 30)	23 (19, 31)	−0.313	0.754
Helplessness	41 (38, 44)	36 (32, 39)	−8.583	<0.001***

*Note:* Categorical variables are presented as case counts (percentages), and continuous variables are presented as median (interquartile range); *Represents *p* < 0.10; ** represents *p* < 0.05; *** represents *p* < 0.001.

### Regression analysis

#### Variable selection and model estimation

**Figure 1.**
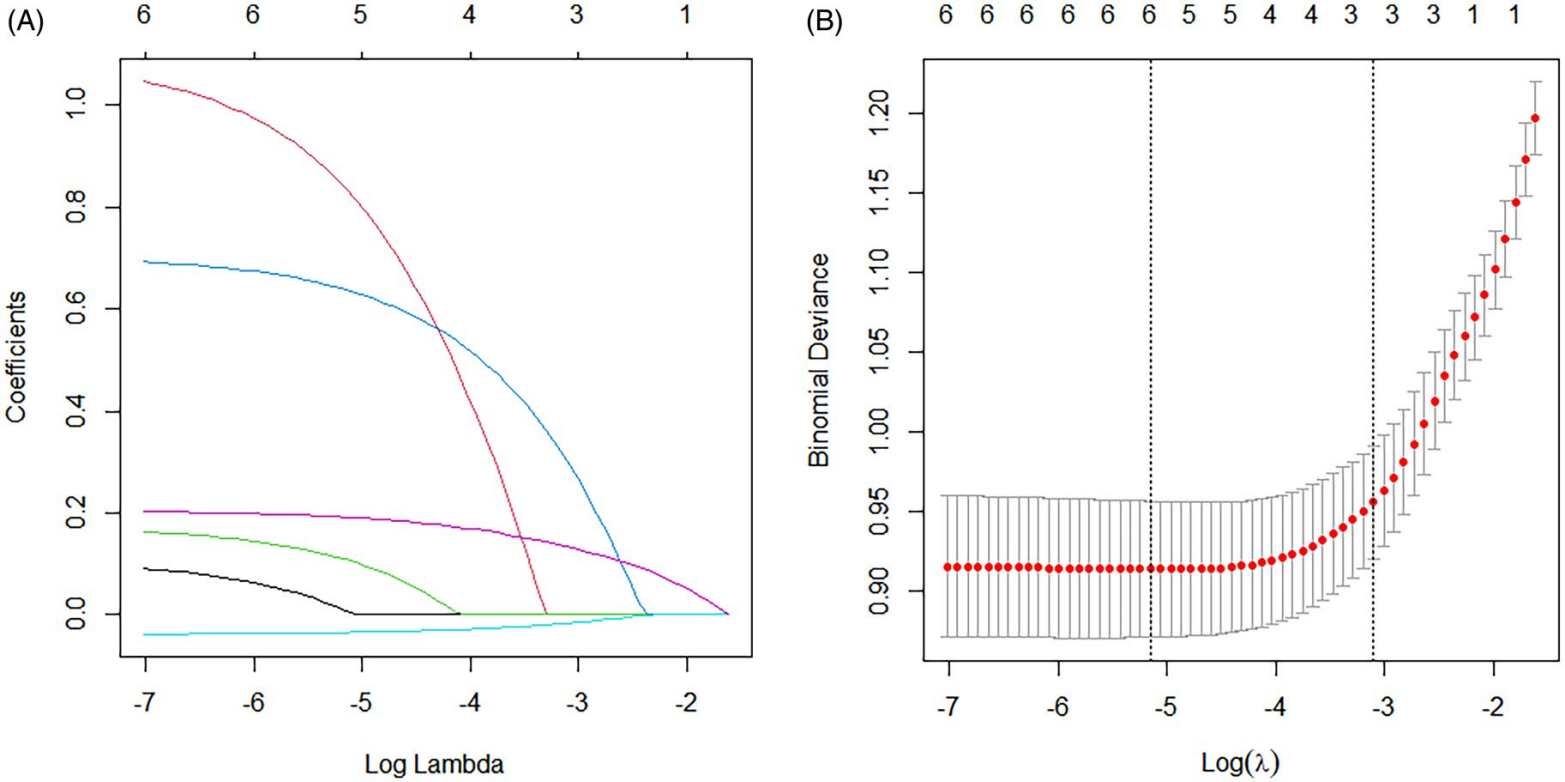
Feature selection based on Lasso regression. A: LASSO coefficient profiles of the six candidate variables. The x‑axis represents log(λ), and the y‑axis represents the values of the regression coefficients. As log(λ) increases, the coefficients are gradually compressed to zero, achieving variable selection. **Figure.** Cross‑validation plot of the LASSO model. The lower x‑axis represents log(λ), the upper x‑axis indicates the number of variables with nonzero coefficients, and the y‑axis shows the mean square error (MSE). The left dashed vertical line (a) corresponds to λ.min (λ = 0.008), the value of λ that minimizes the MSE; the right dashed vertical line (b) corresponds to λ.lse (λ = 0.054), the largest λ within one standard error of the minimum MSE. In this study, λ.lse = 0.054 was selected as the optimal tuning parameter.

Full-variable logistic regression, stepwise logistic regression, and LASSO logistic regression models were built using the six factors with *p* < 0.10 from the univariate analysis as independent variables and medication adherence as the dependent variable. The assignment of each variable is shown in Table 2. Variable selection in the LASSO logistic regression model as λ varies is illustrated in Figure 1. Figure 1A shows the relationship between log(λ) and the LASSO regression coefficients. Figure 1B presents the curve of the number of independent variables plotted against log(λ), with the vertical axis representing the model’s mean square error (MSE), the lower horizontal axis representing log(λ), and the upper horizontal axis indicating the number of non‑zero coefficient independent variables in the model at different log(λ) values. Dashed line a represents the optimal λ.min = 0.008 at which the MSE is minimized, and dashed line b indicates the best λ.lse = 0.054 within one standard error of MSE. This study selected λ.lse = 0.054 as the optimal model, for which the independent variables retained in the LASSO logistic regression model were medication side effects, self‑management, and helplessness.

**Table 2.** Method of assigning values to independent variables.

Characteristics	Symbol	Assignment method
Medication adherence	*Y*	Moderate/high adherence = 1, Low adherence = 2
Marital status	*X*1	Unmarried = 1, Married = 2, Divorce = 3
PD status	*X*2	Continue PD = 1, Do not continue PD = 2, Alternating between PD and HD = 3
PD operator	*X*3	Self = 1, Family = 2
Side effects of medication	*X*4	0-1 = 1, 2–3 = 2, ≥4 = 3
Self-management	*X*5	Substituting the original value
Helplessness	*X*6	Substituting the original value

#### Parameter estimation and model evaluation

**Figure 2.**
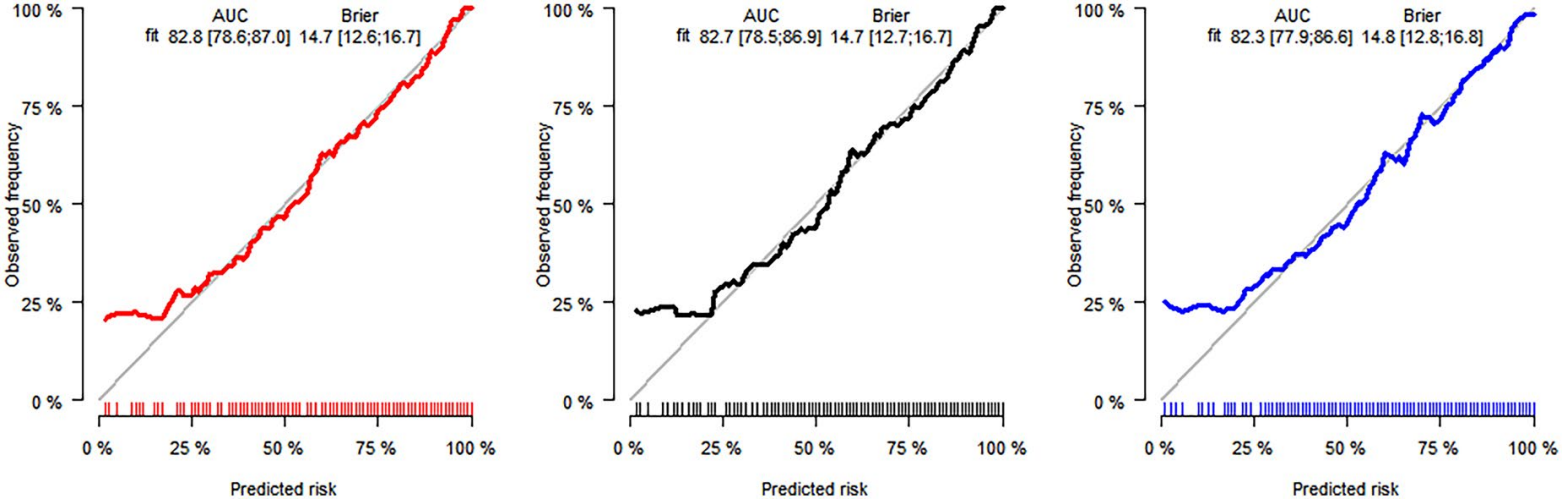
Calibration curves of the three logistic regression models. (A) Full‑variable logistic regression model; (B) Stepwise logistic regression model; (C) LASSO logistic regression model. The x‑axis represents the predicted probability of poor medication compliance, and the y‑axis represents the observed probability. The diagonal dashed line indicates perfect calibration (ideal prediction). The closer the calibration curve aligns with the diagonal line, the better the model calibration. The AUC (area under the curve) and Brier score values are displayed in each panel to evaluate model discrimination and accuracy, respectively.

**Figure 3.**
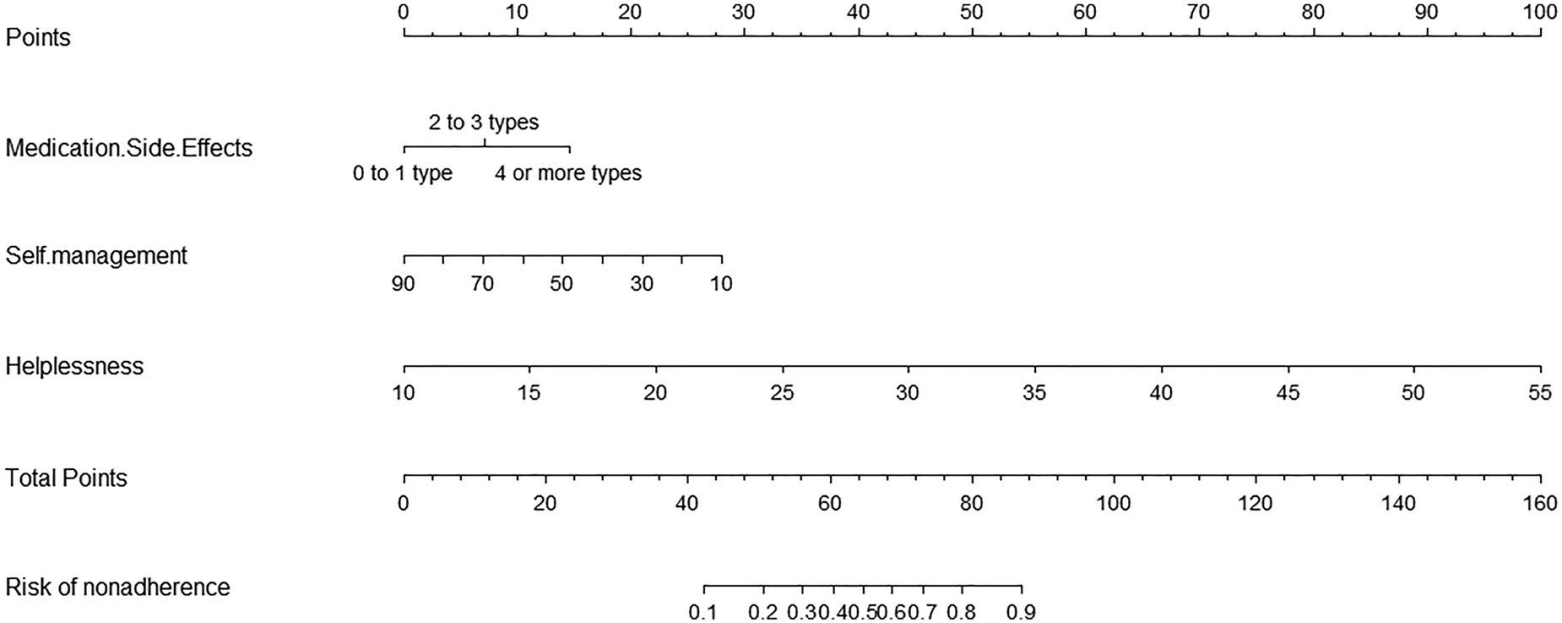
LASSO logistic regression prediction nomograms. The nomogram integrates three predictors: medication side effects, self-management, and helplessness. By summing the points assigned to each predictor, the total points correspond to an estimated probability of poor medication compliance, allowing for individualized risk assessment.

The parameter estimation results of the LASSO logistic regression, full-variable logistic regression, and stepwise logistic regression models are shown in Table 3. The AIC and BIC values were highest for the full‑variable logistic regression model. The AIC of the LASSO logistic regression model was 370.193, which was slightly higher than that of the stepwise logistic regression (368.800), a difference of 1.393. In terms of BIC, the LASSO logistic regression model had a value of 386.169, which was lower than the stepwise logistic regression value of 388.770. This finding suggests that when penalizing for sample size, the overall performance of LASSO regression was better than that of stepwise logistic regression. The calibration curves in Figure 2 show no significant difference in discrimination and accuracy among the three groups, indicating similar predictive power. However, LASSO logistic regression offers the advantages of variable selection and stability, allowing it to accurately identify key influential factors and provide a strong basis for clinical intervention and health management. Its parsimonious structure reduces the burden of data collection and enhances the generalizability and practical utility of the model. Overall, the LASSO logistic regression was chosen as the optimal model in this study. The results of the LASSO logistic regression model showed that medication side effects (*OR* = 2.061, 95%*CI*: 1.368–3.190), self-management (*OR* = 0.962, 95%*CI*: 0.942–0.980), and helplessness (*OR* = 1.226, 95%*CI*: 1.162–1.300) were key factors associated with poor medication adherence among PD patients. The LASSO logistic regression nomogram (Figure 3) provided a visual representation of poor medication adherence among PD patients.

**Table 3.** Results of different regression model analyses.

Variable	Full-variable logistic regression	Stepwise logistic regression	LASSO logistic regression
Marital status	–	–	–
PD status	1.190(0.799,1.827)	–	–
PD operation	2.973(0.971,11.514)*	2.867(9.395,11.070)*	–
Side effects of medication	2.019(1.340,3.122)**	2.026(1.343,3.136)**	2.061(1.368,3.190)***
Self-management	0.962(0.943,0.981)***	0.962(0.943,0.981)***	0.962(0.942,0.980)***
Helplessness	1.227(1.164,1.301)***	1.227(1.163,1.301)***	1.226(1.162,1.300)***
AIC	371.950	368.800	370.193
BIC	399.907	388.770	386.169

*Represents *p <* 0.1; ** represents *p <* 0.01; *** represents *p <* 0.001.

#### Statistical analysis process

**Figure 4.**
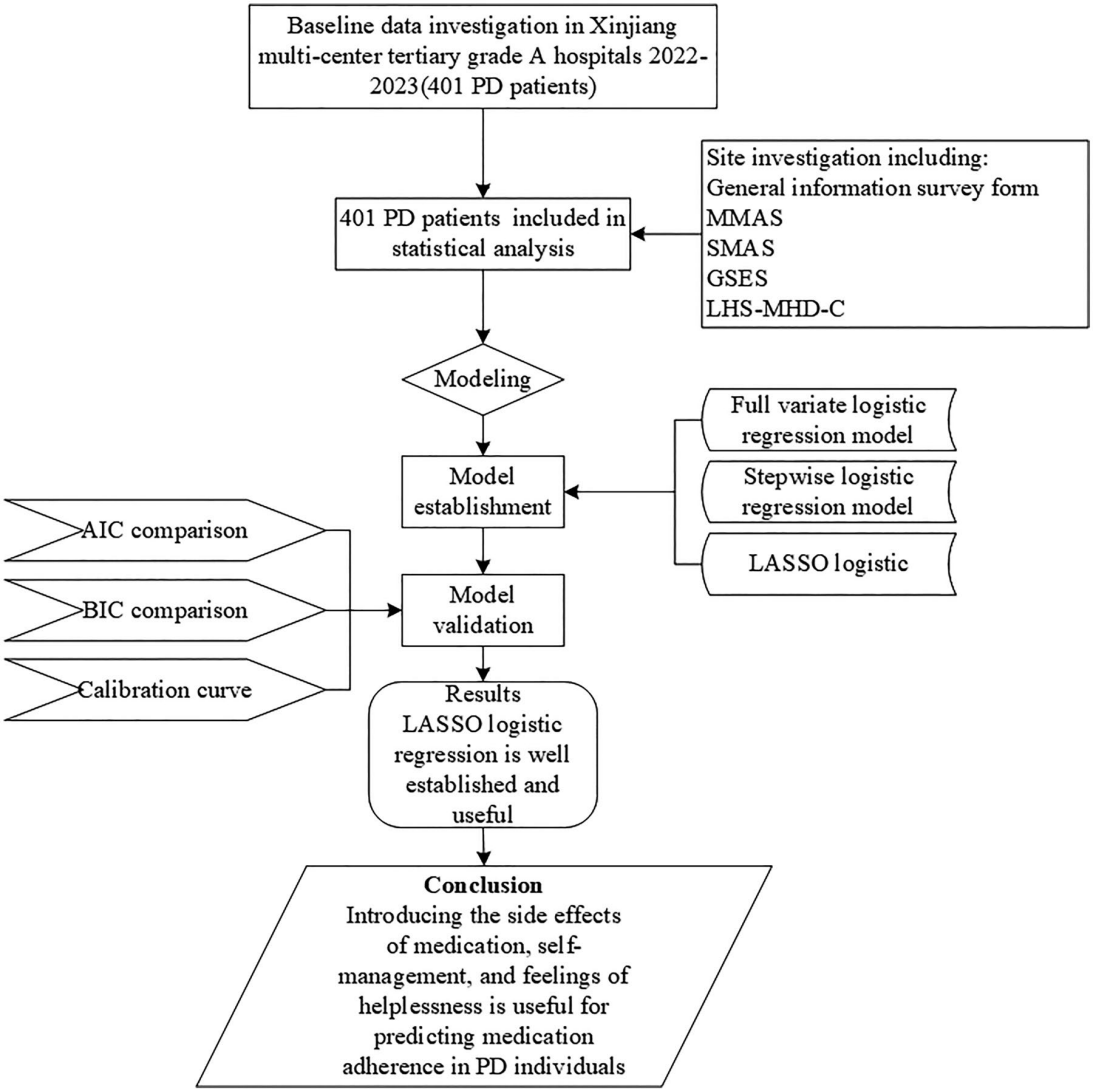
Flowchart of the study design and model development process. Data were collected from 401 maintenance peritoneal dialysis patients across multi‑center tertiary grade A hospitals in Xinjiang from 2022 to 2023. Baseline assessments included general information, the Morisky Medication Adherence Scale (MMAS), Self‑Management Ability Scale (SMAS), General Self‑Efficacy Scale (GSES), and the Lack of Helplessness Scale for Maintenance Hemodialysis Patients‑Chinese version (LHS‑MHD‑C). Three multivariate models—full‑variable logistic regression, stepwise logistic regression, and LASSO logistic regression—were established and compared. The LASSO logistic regression model was selected as the optimal model based on its variable selection capability and stability. The final model identified medication side effects, self‑management, and feelings of helplessness as key factors associated with poor medication adherence in this population.

This study identified six factors associated with medication adherence among PD patients through univariate analysis (*p* < 0.10) and subsequently performed multivariate analysis using the full‑variable logistic regression, stepwise logistic regression, and LASSO logistic regression models. Considering all aspects, LASSO logistic regression was selected as the optimal model because of its advantages in variable selection and stability. Its results showed that medication side effects, self‑management, and helplessness are key factors contributing to poor medication adherence among PD patients (Figure 4).

## Discussion

Medication adherence is a critical determinant of clinical outcomes in patients undergoing PD, yet it remains suboptimal in real‑world settings [8]. In this study, 71.3% of patients exhibited poor medication adherence, a finding consistent with previous reports and reflecting a substantial gap between prescribed therapy and actual patient behavior [21,22]. Given the complex comorbidity profile of PD patients, which includes high rates of cardiovascular disease and diabetes, inadequate adherence may further exacerbate disease progression and increase the risk of adverse outcomes [23].

Using both LASSO and conventional logistic regression approaches, this study consistently identified medication side effects, self-management level, and helplessness as key determinants of poor adherence. Notably, the LASSO model provided a more parsimonious and clinically interpretable framework, supporting its utility for risk stratification in routine practice.

Medication-related side effects emerged as a major barrier to adherence. This finding aligns with prior evidence showing that adverse drug reactions can significantly undermine patients’ willingness to adhere to long-term therapy. In the context of PD, where patients often require complex multidrug regimens, the cumulative burden of side effects may further compromise adherence. Importantly, this issue is not solely pharmacological but also reflects gaps in patient education and communication. Patients who lack an adequate understanding of expected side effects and their management strategies may misinterpret these reactions as treatment failure, leading to intentional non-adherence [24]. Therefore, structured medication counseling, proactive side-effect monitoring, and timely regimen adjustment are essential to mitigate this risk.

Self-management capacity was another significant predictor, underscoring the unique care model of PD. Unlike hemodialysis, PD is largely home-based and requires patients to assume substantial responsibility for treatment implementation, including medication management [25,26]. Insufficient self-management skills may hinder patients’ ability to follow complex therapeutic regimens, particularly in the presence of multiple comorbidities. This finding reinforces the importance of patient-centered education programs aimed at enhancing self-management competencies, which have been shown to improve adherence and overall clinical outcomes in PD populations [8].

Helplessness, as a psychological factor, also played a critical role in medication adherence. Patients undergoing long‑term dialysis frequently experience persistent physical symptoms (such as skin itching, fatigue, headaches, dry mouth, and difficulty sleeping) and treatment burden, which may contribute to negative illness perceptions and emotional distress [27]. Furthermore, from a behavioral perspective, helplessness may reduce patients’ perceived control over their condition and weaken their motivation to engage in self‑care behaviors, including medication adherence. This mechanism is consistent with theoretical frameworks such as the self‑regulation model of chronic illness. The negative disease perceptions resulting from negative emotions in patients with chronic diseases can lead to unhealthy lifestyle habits, such as poor medication adherence [28,29], further influencing their health outcomes and quality of life [30]. Therefore, integrating psychological assessment into routine care and providing targeted interventions, such as cognitive‑behavioral strategies, may help alleviate helplessness and promote adherence. While depression is a widely recognized determinant of adherence in chronic illness, our focus on helplessness was chosen because it directly captures illness‑specific perceptions of control. Future research comparing the predictive power of helplessness versus depression in PD patients would be valuable.

Potential confounding by comorbidities. This study did not assess specific comorbidities, such as diabetes, hypertension, or cardiovascular disease, all of which are common in PD populations. These conditions may independently affect medication adherence by increasing the number of prescribed medications, complicating dosing schedules, and amplifying the burden of side effects. Consequently, the observed associations between medication side effects, self-management, helplessness, and poor adherence could be partially confounded by these unmeasured factors. Future longitudinal studies that systematically collect comorbidity data are warranted to disentangle the independent contribution of each predictor.

From a clinical perspective, the prediction model developed in this study provides a practical tool for early identification of patients at high risk of poor adherence. By focusing on modifiable factors, specifically side effects, self‑management, and psychological status, clinicians can implement targeted, individualized interventions. Such strategies may include optimizing medication regimens to reduce adverse effects, delivering structured self‑management training, and incorporating psychological support into PD care pathways. These measures have the potential to improve adherence, enhance quality of life, and reduce healthcare utilization.

Generalizability considerations. Our study was performed in a single PD center in Xinjiang, China. Although Xinjiang is home to multiple ethnic groups, and our sample included 32.4% minority participants, the adherence rate of 71.3% and the predictive model should be interpreted with caution when applied to other Chinese regions. Healthcare delivery systems, medication accessibility, patient education levels, and cultural norms vary considerably across China. For instance, patients in well‑resourced urban centers may have better access to medication counseling and psychological support, potentially leading to lower rates of nonadherence. Conversely, rural or underdeveloped areas may face even greater challenges. Thus, the specific numerical findings may not be directly transportable; however, the identified risk factors (side effects, poor self‑management, helplessness) are likely to be relevant across settings due to their theoretical foundation. Future research should prioritize multi‑center, cross‑regional studies to test and adapt our LASSO model.

## Limitation

This study has several limitations. First, it only assessed the patients’ medication adherence at a single point in time, failing to capture its dynamic changes. Second, objective clinical indicators, such as biochemical parameters, were not included, which may limit the comprehensiveness of the analysis. Incorporating multidimensional data in future research may provide deeper insights into the mechanisms underlying medication adherence. Third, we did not collect data on specific comorbidities, including diabetes, hypertension, or cardiovascular disease. These conditions are known to influence medication adherence by increasing treatment complexity and pill burden, and their absence may have introduced residual confounding. Fourth, although we collected data on the number of medication types, we used a relatively broad categorization (≤2, 3–4, ≥5 medications) rather than more detailed strata (e.g. 5–9, ≥10). Peritoneal dialysis patients often take 10–15 medications, and a higher pill burden may be associated with worse adherence. Our classification may have underestimated the impact of extreme polypharmacy. Fifth, we assessed only learned helplessness as a psychological factor and did not measure depression. Although helplessness and depression are related constructs, the absence of a validated depression scale limits the comprehensiveness of our psychological evaluation and may affect the generalizability of our findings to settings where depression is routinely assessed. Future studies should include both measures to determine the independent and overlapping effects of helplessness and depression on adherence in PD patients.

## Conclusion

Poor medication adherence is highly prevalent among patients undergoing maintenance PD. Medication side effects, low self‑management capacity, and elevated helplessness are key modifiable risk factors. Targeted interventions addressing these domains may play a pivotal role in improving adherence and optimizing patient outcomes.

## Relevance for clinical practice

The findings of this study have direct implications for clinical practice. First, individualized medication management strategies, including dose adjustment and regimen optimization, are essential to minimize side effects and improve tolerability [31]. Second, routine assessment of psychological status, particularly helplessness, should be integrated into PD care, with appropriate interventions such as cognitive‑behavioral therapy [32] when indicated, to help patients establish a positive mindset and enhance their self‑management abilities. Third, medication adherence can be improved through education and training that strengthen patients’ self‑management skills and enable them to understand and manage their illness [33]. In summary, these measures will help increase patient medication adherence, reduce disease‑related complications, improve quality of life, and lower healthcare costs.

## Supplementary Material

Questionnaire.docx

## Data Availability

The data that support the findings of this study are available from the corresponding author Dr. Li Li upon reasonable request.
