# Supplementary material for: Predictors of poor medication adherence in patients undergoing peritoneal dialysis: a LASSO-based risk model from a cross-sectional study in Xinjiang, China
Source: Ren Fail. 2026 Jun 24;48(1):2687196. doi: 10.1080/0886022X.2026.2687196 (PMC13307385; doi:10.1080/0886022X.2026.2687196)
Supplement: Questionnaire.docx [file IRNF_A_2687196_SM7537.docx]

**Peritoneal Dialysis Patient Medication Adherence Questionnaire**

Dear friend: Hello! To understand your health condition and help medical staff provide more suitable treatment and health education, we are conducting this survey. The information you provide will be kept strictly confidential. Please answer truthfully. Thank you for your support.

**Part 1: General Information**

A1. Your gender? [Single Choice]

①Male ②Female

A2. Your age? [Fill-in-the-blank] :_________

A3. Your education level :[Single Choice]

①Primary school (≤6 years)

②Middle school (including vocational school, 7-9 years)

③University (≥10 years)

A4. Your marital status? [Single Choice]

1. Single ②Married ③Divorced

A5. Your ethnicity? [Single Choice]

1. Han ②Minority

A6. Your employment status? [Single Choice]

1. Employed ②Unemployed

A7. Your medical insurance type? [Single Choice]

①Self-pay

②Urban medical insurance

③New rural cooperative medical insurance

A8. Your household registration type? [Single Choice]

①Rural ②Urban

A9. Have you experienced peritoneal infection? [Single Choice]

①Yes ②No

A10.Who performs your peritoneal dialysis? [Single Choice]

①Yourself ②Family member ③Caregiver/nanny

A11.How many months have you been on peritoneal dialysis? (If less than 1 month, count as 1 month) [[Fill-in-the-blank] ：________

A12.What is your current peritoneal dialysis treatment status? [Single Choice]

①Continuing peritoneal dialysis

②No longer on peritoneal dialysis

③Alternating between peritoneal dialysis and hemodialysis

A13.How many types of medications do you take daily? [Single Choice]

① ≤2 types ②3-4 types ③≥5 types

A14.How many types of side effects do you experience while taking medications? [Single Choice]

①0-1 type ②2-3 types ③4 or more types

A15.How many times do you get hospitalized on average per year? [Single Choice]

①1-2 times per year ②3-5 times per year ③6 or more times per year

A16.Do you wish to receive health guidance related to medications? [Single Choice]

1. Yes ②No

**Part 2: Medication Adherence (C-MMAS-8)**

Please answer the following questions based on your actual situation: [Single Choice]

B1. Do you sometimes forget to take your medication?

①Yes ②No

B2. In the past 2 weeks, have you forgotten to take your medication for one or more days?

1. Yes ②No

B3. During treatment, when you feel worse after taking medication, do you reduce the dose or stop taking it without consulting your doctor?

1. Yes ②No

B4. When you travel or are away from home for a long time, do you sometimes forget to bring your medication?

1. Yes ②No

B5. Did you take your medication yesterday?

1. Yes ②No

B6. When you feel your condition is under control, do you sometimes stop taking your medication?

1. Yes ②No

B7. Taking medication daily can be inconvenient for some people. Do you find it difficult to stick to your treatment plan?

1. Yes ②No

B8. Do you find it hard to remember to take all your medications on time and in the correct dosage?

1. Never ②Occasionally ③Sometimes ④Often

**Part 3: Helplessness (LHS-MHD-C)**

The following questions reflect your level of helplessness since starting treatment. Please answer truthfully. [Single Choice]

C1. My condition has severely affected my life.

①Strongly disagree ②Disagree ③Agree ④Strongly agree

C2. Managing my condition is largely my own responsibility.

①Strongly disagree ②Disagree ③Agree ④Strongly agree

C3. Changes in my condition often catch me off guard.

①Strongly disagree ②Disagree ③Agree ④Strongly agree

C4. If I can manage myself well, I can successfully control my condition.

①Strongly disagree ②Disagree ③Agree ④Strongly agree

C5. I can make a lot of efforts to control my condition.

①Strongly disagree ②Disagree ③Agree ④Strongly agree

C6. When I manage my personal life well, my condition does not worsen.

①Strongly disagree ②Disagree ③Agree ④Strongly agree

C7. I have considerable ability to control my condition.

①Strongly disagree ②Disagree ③Agree ④Strongly agree

C8. I would feel helpless if I couldn’t rely on others to help manage my condition.

①Strongly disagree ②Disagree ③Agree ④Strongly agree

C9. No matter what I do, my condition seems impossible to improve.

①Strongly disagree ②Disagree ③Agree ④Strongly agree

C10. I can effectively cope with changes in my condition.

①Strongly disagree ②Disagree ③Agree ④Strongly agree

C11. It seems like many uncontrollable factors affect my condition—maybe it’s just my fate.

1. Strongly disagree ②Disagree ③Agree ④Strongly agree

**Part 4: Self-Management (SMAS)**

The following questions are about your self-management. Please select the option that best matches your actual situation. [Single Choice]

D1. Check the expiration date, concentration, clarity, and integrity of the dialysis fluid bag, and warm the dialysis fluid.

① Always ②Often ③Occasionally ④Never

D2. Correctly connect and drain the effluent.

① Always ②Often ③Occasionally ④Never

D3. Correctly vent and flush the tubing.

① Always ②Often ③Occasionally ④Never

D4. Correctly infuse the dialysis fluid and disconnect the tubing.

① Always ②Often ③Occasionally ④Never

D5. Avoid touching the short tube connector and double-bag connector to prevent contamination during iodine cap replacement.

① Always ②Often ③Occasionally ④Never

D6. Care for the exit site: clean in a circular motion from the inside out, ensuring disinfectant does not enter the exit site or tunnel.

① Always ②Often ③Occasionally ④Never

D7. Follow the doctor’s instructions for using dialysis fluid.

① Always ②Often ③Occasionally ④Never

D8. If the short tube leaks or falls off, close the proximal end of the dialysis tube, pause dialysis, cover the area with sterile gauze, and seek medical attention to replace the short tube.

① Always ②Often ③Occasionally ④Never

D9. If drainage is poor, check if the switch is open, if the tubing is compressed or twisted, if there is fibrin blockage, and adjust posture or ensure bowel movements.

① Always ②Often ③Occasionally ④Never

D10. If the short tube spiral connector is contaminated, immediately close the short tube.

① Always ②Often ③Occasionally ④Never

D11. If the tubing disconnects or leaks during infusion, stop the infusion, drain the fluid that has entered the abdominal cavity, and contact the dialysis nurse.

① Always ②Often ③Occasionally ④Never

D12. Eat less plant protein and consume an appropriate amount of high-quality protein.

① Always ②Often ③Occasionally ④Never

D13. Eat less high-phosphorus food and choose high- or low-potassium foods based on electrolyte levels.

① Always ②Often ③Occasionally ④Never

D14. Consume an appropriate amount of salty and sodium-containing foods.

① Always ②Often ③Occasionally ④Never

D15. Adjust fluid intake based on ultrafiltration volume, edema, and urine output.

① Always ②Often ③Occasionally ④Never

D16. Eat less high-fat food.

① Always ②Often ③Occasionally ④Never

D17. Measure blood pressure regularly.

① Always ②Often ③Occasionally ④Never

D18. Weigh yourself every morning and monitor for lower limb and eyelid edema.

① Always ②Often ③Occasionally ④Never

D19. Follow the doctor’s instructions for regular peritoneal assessments and short tube replacement.

① Always ②Often ③Occasionally ④Never

D20. Monitor for symptoms such as limb weakness, numbness in hands, feet, or around the mouth.

① Always ②Often ③Occasionally ④Never

D21. Monitor for chest pain, shortness of breath, palpitations, and worsening fatigue.

① Always ②Often ③Occasionally ④Never

D22. Monitor for skin itching.

① Always ②Often ③Occasionally ④Never

D23. Monitor for abnormal bulges in the abdomen or groin.

① Always ②Often ③Occasionally ④Never

D24. Monitor sleep quality.

① Always ②Often ③Occasionally ④Never

D25. When experiencing negative emotions, seek help from family, friends, or the dialysis nurse.

① Always ②Often ③Occasionally ④Never

D26. Avoid anxiety, regulate emotions, and live a peaceful and happy life.

① Always ②Often ③Occasionally ④Never

D27. Complete and enjoy doing things within your ability.

① Always ②Often ③Occasionally ④Never

D28. Socialize with friends, attend gatherings, and participate in recreational activities.

1. Always ②Often ③Occasionally ④Never

**Part 5: Self-Efficacy (GSES)**

The following questions are about your confidence. Please select the option that best matches your actual situation. [Single Choice]

E1. If I try hard enough, I can always solve problems.

①Not at all true ②Slightly true ③Mostly true ④Completely true

E2. Even if others oppose me, I can still find a way to get what I want.

①Not at all true ②Slightly true ③Mostly true ④Completely true

E3. I am confident that I can effectively handle any unexpected events.

①Not at all true ②Slightly true ③Mostly true ④Completely true

E4. It is easy for me to stick to my ideals and achieve my goals.

①Not at all true ②Slightly true ③Mostly true ④Completely true

E5. With my intelligence, I can handle unforeseen situations.

①Not at all true ②Slightly true ③Mostly true ④Completely true

E6. If I put in the necessary effort, I can solve most problems.

①Not at all true ②Slightly true ③Mostly true ④Completely true

E7. I can calmly face difficulties because I trust my ability to handle problems.

①Not at all true ②Slightly true ③Mostly true ④Completely true

E8. When facing a problem, I can usually find several solutions.

①Not at all true ②Slightly true ③Mostly true ④Completely true

E9. When in trouble, I can usually think of ways to cope.

①Not at all true ②Slightly true ③Mostly true ④Completely true

E10. No matter what happens to me, I can handle it.

①Not at all true ②Slightly true ③Mostly true ④Completely true
